# Mechanisms and therapeutic prospects of mesenchymal stem cells-derived exosomes for tendinopathy

**DOI:** 10.1186/s13287-023-03431-3

**Published:** 2023-10-26

**Authors:** Yuxiang Zhang, Wei Ju, Hong Zhang, Liu Mengyun, Weiliang Shen, Xiao Chen

**Affiliations:** 1 Present Address: Department of Plastic Surgery, Sir Run Run Shaw Hospital, Zhejiang University School of Medicine, Hangzhou, China; 2 Dr. Li Dak Sum & Yip Yio Chin Center for Stem Cell and Regenerative Medicine and Department of Orthopedic Surgery of Sir Run Run Shaw Hospital, Zhejiang University School of Medicine, Hangzhou, China; 3 Key Laboratory of Tissue Engineering and Regenerative Medicine of Zhejiang Province, Zhejiang University School of Medicine, Hangzhou, China; 4 Department of Sports Medicine, Zhejiang University School of Medicine, Hangzhou, China; 5 China Orthopedic Regenerative Medicine Group (CORMed), Hangzhou, China; 6 Dr. Li Dak Sum-Yip Yio Chin Center for Stem Cells and Regenerative Medicine and Department of Orthopedic Surgery of The Second Affiliated Hospital, Zhejiang University School of Medicine, Hangzhou, China

**Keywords:** Tendinopathy, Exosomes, Mesenchymal stem cells, Therapeutics

## Abstract

Tendinopathy is a debilitating and crippling syndrome resulting from the degeneration of tendon tissue, leading to loss of mechanical properties and function, and eventual tendon rupture. Unfortunately, there is currently no treatment for tendinopathy that can prevent or delay its progression. Exosomes are small extracellular vesicles that transport bioactive substances produced by cells, such as proteins, lipids, mRNAs, non-coding RNAs, and DNA. They can generate by mesenchymal stem cells (MSCs) throughout the body and play a role in intercellular communication and regulation of homeostasis. Recent research suggests that MSCs-derived exosomes (MSCs-exos) may serve as useful therapeutic candidates for promoting tendon healing. This review focuses on the function and mechanisms of MSCs-exos in tendinopathy treatment and discusses their potential application for treating this condition.

## Introduction

Tendinopathy refers to a multifaceted disease with symptoms including discomfort, malfunction, and exercise resistance decrease and comprise 30% of referrals to musculoskeletal physicians [1]. Specifically, the prevalence of tendinopathy is 15% among elite athletes [2] and 30–50% among people over sixty [3]. Tendinopathy is associated with high morbidity with complex etiologies; however, its pathogenesis remains unknown. There is consensus that exogenous factors, such as mechanical overload, and/or endogenous factors, such as dysregulated apoptosis, disrupt the balance between matrix metalloproteinases (MMPs) and corresponding inhibitors and growth factors [4, 5], leading to inflammation and degeneration. Low-level chronic tendinitis may result from mechanical overburden, which is likely involved in the pathogenesis of tendinopathy [6]. These complex pathological changes present a challenge in developing effective treatments for tendon healing. While conservative, first-principles treatments for tendinopathy such as rest, physiotherapy, and medicine may relieve symptoms, they do not address the underlying pathology because of the short duration of disease-modifying therapeutics [7]. Although surgical therapies are a final resort, outcomes from surgery are unsatisfactory due to high rates of complications such as adhesion and rupture [8]. Consequently, the development of new treatment strategies for tendinopathy is urgent.

Due to the unique differentiation potential and self-renewal ability, MSCs have been widely used in the treatment of wound, fibrosis, and other proinflammatory diseases. MSCs-based treatment for tendinopathy is a promising method that has been developing further in recent years. Preclinical trials have shown that the direct injection of MSCs derived from adipose tissue or bone marrow can protect the tendon from degeneration and delay the progression of tendinopathy [9, 10]. Additionally, MSC-based therapies for tendinopathy have been shown to reduce inflammation and discomfort in several clinical trials [11]. Nevertheless, some studies have announced the deficiency of MSCs implantation. The implantation of MSCs isolated from bone marrow, for example, can result in teratomas [12]. As such, researchers are endeavoring to investigate an effective approach for utilizing MSCs as safe and efficacious therapies.

Examples of these efforts include therapeutic amplification, genomic modification, drug combination, and the use of exosomes. Exosomes are a type of vesicle in size range smaller than 200 nm [13]. As a subtype of extracellular vesicles, exosomes are originated from the outward budding of the plasma membrane and intracellular endocytic trafficking pathway [13]. One advantage of using exosomes for treatment is that these vesicles have been developed to avoid the side effects of cell therapy. Recent research has shown that MSCs influence recipient cells mainly by secreting large amounts of exosomes. Exosomes have the potential to improve collagen production and angiogenesis by increasing mRNA expression and releasing proangiogenic stimuli factors and regulatory proteins. ADSCs-exosomes (ADSCs, adipose-derived mesenchymal stem cells) had more beneficial effects on tendon repair than ADSCs-ectosomes in Achilles tendinopathy, this partly attribute to mRNA expression difference [14].

This review attempts to summarize the current status of the use of exosomes for the treatment of tendinopathy and introduce the detailed roles that exosomes play in each pathological process of tendinopathy. Additionally, we will prospect the potential of exosome-based therapeutics for tendinopathy patients in the future.

## Origin and development of exosomes

The exosome was first discovered in 1983 by Harding in the maturing mammalian reticulocyte [15]. Its transportation was later found to be through exocytosis and transferrin receptors [16]. Following that, Raposo et al. found that exosomes strengthened antigen presentation and T-cell activation and thus enhanced immunity [17]. The primary function of exosomes in tumors was unveiled since exosomes were found to produce a spectrum of molecules involved in immune responses and signal transductions [18]. With the accumulation of further studies, the delicate balance in cell interactions driven by exosomes was revealed. Since Valadi et al. reported that exosomes can facilitate intercellular contact via nucleic acid (mRNA and microRNA) delivery in 2007, numerous studies have verified the indispensable role of exosomes in mediating intercell connectivity [13, 19, 20]. Further study revealed that exosomes contribute to yet more advantages, such as improved circulation stability and biocompatibility, without immunogenicity and toxicity [21]. Exosomes, as a signaling modality, generally bear the distinct contents of their parental cells and modulate gene expression in recipient cells by releasing their protein, RNA, and other molecules to the recipient cells, thereby producing a diverse range of biological effects. In addition, they are feasible to mass-produce and can be applied directly instead of via engrafting, which avoids a common obstacle in traditional cell-based therapy [22]. Given these advantages, exosomes may be an effective therapeutic alternatives for tendinopathy.

### Biogenesis of exosomes

**Fig. 1:**
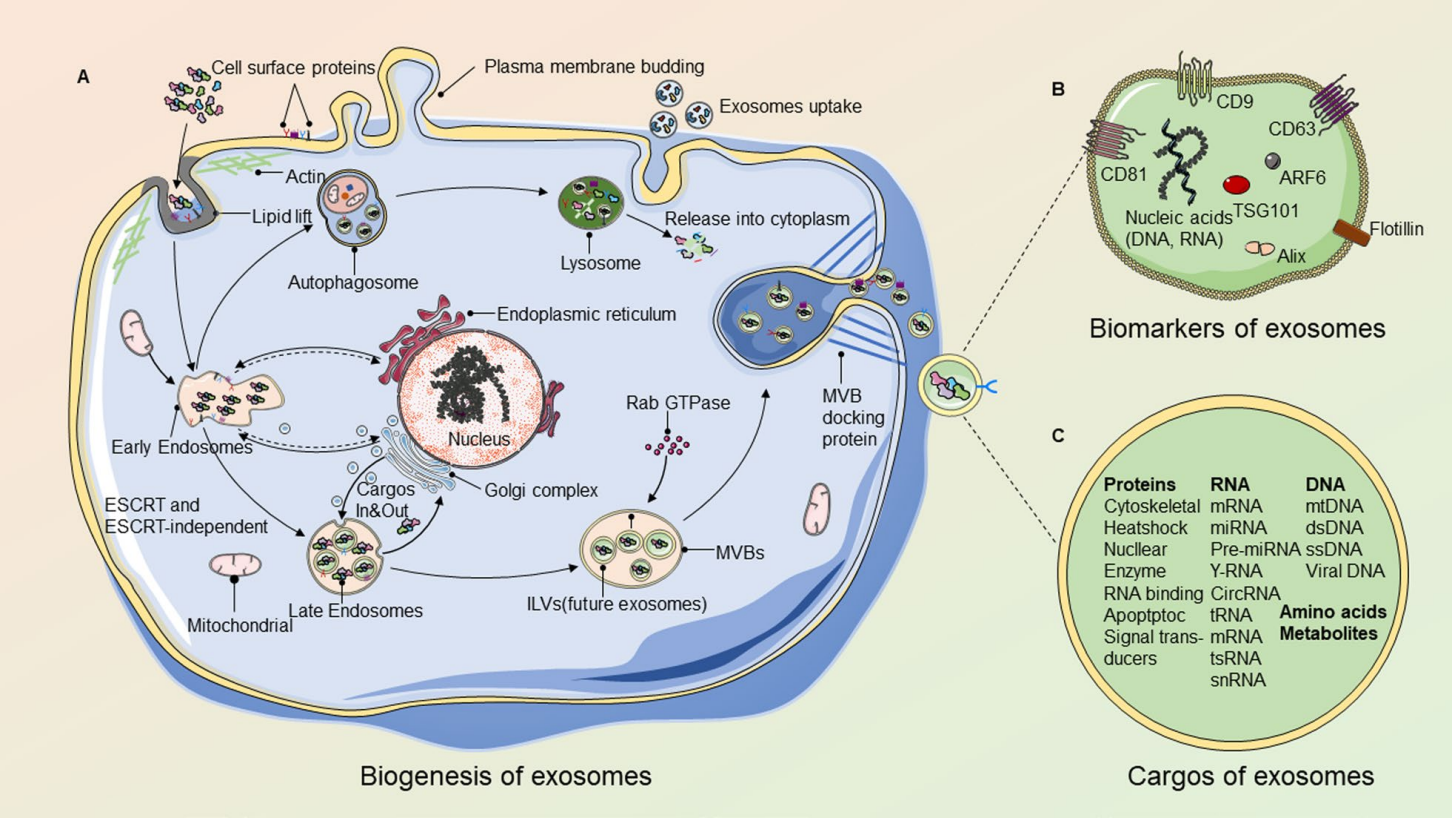
Biogenesis, biomarkers, and cargos of exosomes. **A** Endocytosis and plasma membrane invagination enable extracellular constituents and cell surface proteins to invade cells. Early endosomes are formed when a plasmalemma buds and fuses with the endoplasmic reticulum, trans Golgi body, and mitochondrial constituents. Then, late endosomes are formed, which undergo a second invagination by cargo modification, resulting in the generation of various ILVs and the development of MVBs. The majority of MVBs will then be transferred to the plasmalemma and dock on the inner face, while others fuse with lysosomes via autophagosomes, causing their contents to be degraded. Finally, MVBs release ILVs to the outside of the cell through exocytosis and become exosomes. Exosomes originating from other cells may also be taken up by the cell in the meantime [28]. **B** Exosomes include a variety of nucleic acids, amino acids, proteins, and metabolites. Rab GTPases, ESCRT proteins (see text), and other proteins that are often recognized as exosome biomarkers are among the proteins implicated in exosome biogenesis (CD9, CD81, CD63, flotillin, TSG101, ceramide, and Alix). Proteins on the surface of exosomes include tetraspanins, integrins, and immunomodulatory proteins [26]. **C** Exosomes may also contain intracellular proteins, RNA, DNA, amino acids, and metabolites, among other things [34]. (Note: This figure was created by the authors and there is no confliction of copyright.)

Exosomes, which originate from the endosomal system or are released by shedding from the plasma membrane, are membrane-bound structures that offer a unique mechanism of transcellular communication through their release and uptake. While the function of exosomes is determined by the properties of their parent cells, the biogenesis and release processes of exosomes are common to nearly all cells [23]. The biogenesis of exosomes requires several stages and takes various forms. Although much of their complex biogenesis remains unknown, the mechanisms underlying the formation of exosomes have been identified with the progressive study. In the beginning, endocytic vesicles arise from the plasma membrane through lipid raft domains through endocytosis, contributing to early intracellular endosome formation. The Golgi apparatus is involved in the transition of endosomes from early to late endosomes and collects intraluminal vesicles (ILVs) in the lumen at the same time [24]. Early endosomes can either return to the plasmalemma or be inserted into ILVs [25]. Endocytic sorting of complex-mediated cargo into ILVs requires endosomal sorting complex required for transport (ESCRT)-dependent and ESCRT-independent pathways [26]. These ILVs accumulate in late endosomes through the inward budding and cytosol sequestration of the early endosomal membrane [27]. This process transforms endosomes into multivesicular bodies (MVBs). Later, these MVBs fuse with lysosomes (resulting in the degrading of the ILV) or plasma membranes (resulting in the release of exosomes) [28]. Rab guanosine triphosphatase (GTPase) proteins regulate MVBs transportation and fusion, and cytoskeletal and molecular motor are also involved in this process [29, 30]. However, the mechanisms that control whether MVBs migrate to lysosomes or the plasma membrane and the hidden pathways that control exosome secretion are still poorly understood. Current research suggests that various subpopulations of MVBs may exist in cells at the same time, implying that some are destined for degradation, while others are destined for exocytosis [31] (Fig. 1A).

### Molecular vehicles

The composition of exosome membranes is characterized by lipid rafts. In contrast to their parental cells, exosomes have higher levels of specific lipid species including sphingomyelin, phosphatidylcholine, cholesterol, ceramide, and diacylglycerol [32, 33] (Fig. 1B-C).

The molecular components within the exosomes vary and are affected by a variety of factors. It is evident that the function of exosomes is largely determined by its contents, which are closely related to the properties of their parent cells [23]. Pefanis et al. have discovered that exosomes contain several types of RNA, such as miRNAs, mRNAs, tRNAs, lncRNAs, and rRNAs [34]. Different types of RNA cargo can play a specific role in cell epigenetic alteration and biological activity. Recent research has found that the mutational status of tumors could be determined by dsDNA found in tumor-derived exosomes [33, 35]. In addition to nucleic acids, bioactive proteins that originated in the cytoplasm have also been found in exosomes, including those involved in the biogenesis of exosomes [35]. Exosomes are also able to convey proteins involved in intracellular assemblage and trafficking, such as tetraspanins, heat shock proteins, and integrins [35]. The contents MSCs-exos have a wide range of potential applications due to their ability to promote cell proliferation, cell differentiation, anti-inflammatory responses, and anti-aging effects. These effects make them particularly promising for the treatment of various conditions, including but not limited to cardiovascular diseases, neurological disorders, and skeletal muscle diseases.

## The function of MSCs-exos in tendinopathy

Due to their varying contents, exosomes generated by different tissues affect a variety of cellular properties. Therefore, decoding the tissue-specific contents of exosomes is pivotal in understanding how these vehicles may affect a target cell. MSCs-exos have been shown to regulate the phenotype and function of specific cells via the nucleic acids and proteins they contain [36].

### Maintain a homeostasis of tendon under hypoxia

Hypoxia has been identified to be the priming signal to initiate the molecular pathology of rotator cuff tendinopathy [37]. Intense hypoxia affects the vascularity of the tendon, causing apoptosis and necrosis of tenocytes and thus aggravating tendinopathy [38]. The hypoxic environment also initiates the MSC response and alleviates the tendon injury. It has shown that hypoxic conditions (94% N_2_, 5% CO_2_, and 1% O_2_) can stimulate the migration and proliferation of MSCs and also promote the secretion of anti-inflammatory factors, which can contribute to tendon repair [39]. As a vehicle, exosomes may be involved in the secretion and delivery of these factors, while MSCs regulate the tissue cell response to hypoxia through exosomes. This suggests that exosomes derived from hypoxic tissue cells, especially MSCs in the hypoxia niche, may play a positive role in the treatment of tendinopathy [40]. MSCs regulate tissue cells’ response to hypoxic environments by the contents of exosomes. Tendon stem cells (TSCs) were first identified in humans and mice in 2007, and they are closely related to BMSCs but not identical [41]. As a type of MSCs, TSCs represent a more appropriate cell source for the regeneration of musculoskeletal tissue, particularly tendon tissue [42]. Finosh et al. investigated the factors and proteins delivered by exosomes derived from swine hypoxia TSCs. Mass spectrometry analysis showed that synthesis of COLA12, PDIA4, COLG, FN1, CTSK, and TN-C was downregulated, while COL1A2, P4HA1, PRDX2, P3H1, COL6A1, PPIB, LCN1, and COL3A1 was upregulated. With network analyst, these proteins were revealed to interact with different kinds of proteins and the most essentially, control several pathways associated with ECM homeostasis and repair [37]. The contents of exosomes derived from TSCs and subcutaneous ADSCs cultured in hypoxia were also analyzed. MMP2, COL6A, CTSD, and TN-C were the primary proteins regulate ECM homeostasis in hypoxia ADSCs, while THSB1, NSEP1, ITIH4, and TN-C regulated tenocytes homeostasis in hypoxia [43]. While these proteins are active in a variety of ECM repair mechanisms, they are also active in regeneration signaling pathways, suggesting that downstream ECM regenerative mechanisms should be investigated next. Regardless, the regenerative mediators in exosomes derived from TSCs/ADSCs in response to hypoxia provide fresh translational potential for tendinopathy treatment.

### Regulation of the immune microenvironment in tendinopathy

**Fig. 2:**
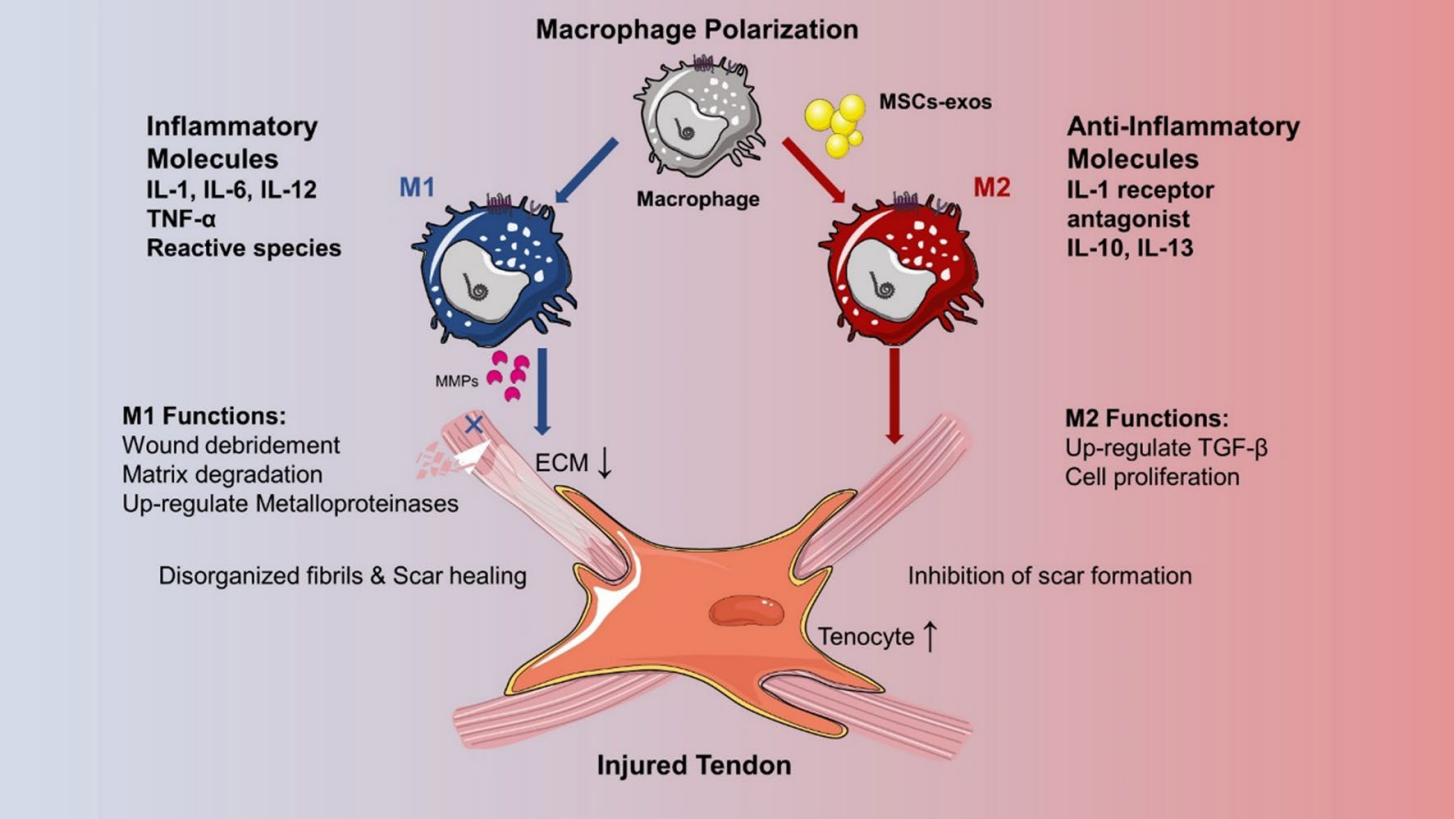
Macrophages can be induced to an activated inflammatory phenotype by exosomes secreted by MSCs/TSCs. Exosomes regulate the inflammation process after tendon injury to facilitate proper healing through M1 down-regulation and M2 up-regulation, which alleviate inflammation and determine whether repair or degeneration will occur [46–48]. (Note: This figure was created by the authors and there is no confliction of copyright.)

The immune system, as a defense system of an organism, protects against the invasion of pathogens. Additionally, the immune system can regulate tissue development, homeostasis, and repair processes. Specifically, MSCs-exos have the potential to suppress the inflammation response in early injury and facilitate tissue repair [44]. By suppressing the early inflammatory response in tendon injury, MSC-exos can affect healing directly [10]. Shen et al. investigated the effect of extracellular vehicles (EVs) generated by ADSCs on regulating tissue responses in early tendon healing and discovered that ADSC-EVs are able to regulate the macrophage inflammatory response by blocking NF‐κB activity, whose pathway has also confirmed to have protective effect in tendinopathy [10, 45]. The polarization of macrophages transforms macrophages into one of two phenotypes: the M1-like phenotype, which has a proinflammatory effect or the M2-like phenotype, which has an anti-inflammatory effect, and is among the innate immune responses. The phenotype transformation of M1 to M2 would facilitate tissue repair (Fig. 2). ADSCs-derived exosomes have been shown to improve the histological characteristics as well as biomechanical strength by enhancing M2 polarization in chronic rotator cuff (RC) tendinopathy [46]. As such, controlling inflammation following a tendon injury is critical for promoting high-quality healing. TSCs-derived exosomes (TSCs-exos) can significantly decrease the number of CCR7^+^M1 macrophages and significantly increase the number of CD163^+^M2 macrophages. Similarly, in another study, IL-10 (M2 promoting growth factor) expression was observed to be higher, while IL-6, an inflammatory cytokine, was down [47]. Macrophages were treated with exosomes isolated from MSCs to create exosome‐educated macrophages (EEMs). These exogenous EEMs were found to strengthen tendon mechanical properties, facilitating angiogenesis and showing marked suppressive effects on inflammation, improving healing [48]. However, the underlying mechanisms of the anti-inflammatory effects have yet to be established. Hence, the immunomodulatory function of exosomes should be further considered.

### Promote proliferation and migration of tenocytes

**Fig. 3:**
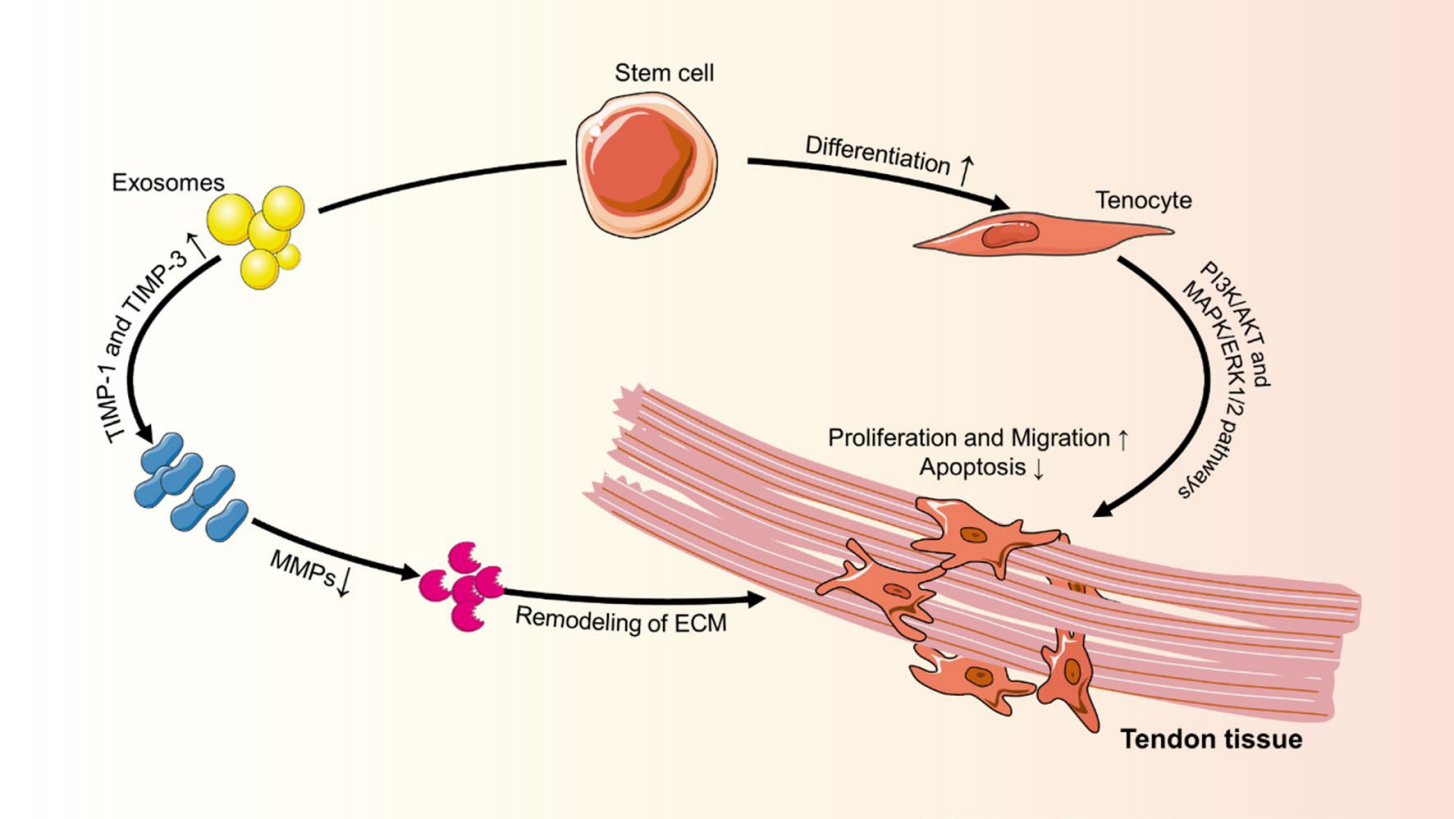
Proliferation and migration of tenocytes can be promoted by exosomes in a dose-dependent manner via PI3K/AKT and MAPK/ERK1/2 signaling pathways. Exosomes also decrease the apoptosis of tenocytes. MMP and TIMP released by exosomes are the most important factors in aiding the healing process. TSCs-exos reduce MMP-3 and MMP-9 expression while increasing expression of metalloproteinase inhibitors (TIMP-1, TIMP-3) and improve tendons' mechanical properties. (Note: This figure was created by the authors and there is no confliction of copyright.)

In tendons, the primary resident cells are tenocytes. The proliferation and migration of TSCs and tenocytes are important for the maintenance of tendon integrity, remodeling, and repair. TSCs-exos may facilitate the tenocytes’ proliferation and migration, which functions in a dose-dependent manner via the PI3K/AKT and MAPK/ERK1/2 signaling pathways and decreases tenocyte apoptosis [47]. Bone marrow mesenchymal stem cells-derived exosomes (BMSCs-exos) perform at similar levels to TSCs-exos and are able to enhance TSCs tenogenic differentiation [49]. Enrichment of TSCs-exos by transforming growth factor (TGF) effectively accelerates tenocyte proliferation and migration via activation of the TGF-Smad2/3 and ERK1/2 signaling pathways in TSCs [50] (Fig. 3). These studies facilitate a deeper comprehension of the interaction between MSCs-exos and TSCs or tenocytes and provide a theoretical foundation for the regeneration and repair of tendon injuries.

### Promote the synthesis of extracellular matrix

The extracellular matrix The extracellular matrix (ECM) of tendon/ligament is mostly composed of aligned collagen I, which gives it its structure and mechanical properties, while type III collagen accounts for only 10% of the total collagen in the tendons [51]. Several studies have established a connection between ECM disorders and the pathogenesis of tendinopathy. Collagen degradation occurs at a faster rate than collagen synthesis in tendinopathy. MMPs and tissue inhibitors of metalloproteinases (TIMP) play a crucial role in ECM turnover and remodeling, and an imbalance between the two may result in the destruction of tendon microstructure and composition [52]. In tendon healing, collagen fibers with a larger diameter are mechanically stronger than fibers with smaller diameters; therefore, collagen fiber size contributes to the mechanics of tendon repair [53]. Additionally, the ratio of type I/III collagen is critical to effective tendon healing [47]. TSCs-exos and ADSCs-exos have been shown to balance ECM components and increase the ratio of large-diameter to small-diameter fibrils. This improved biomechanical properties of the tendon by improving the type I/III collagen ratio [14, 47]. The exosomes derived from tendon stem cells demonstrated a significant reduction in MMP-3 and an increase in the expression of regulatory proteins such as Col-1a1 and TIMP-3 in vitro. In vivo injection of these exosomes led to a marked decrease in the expression of MMP-3 and an elevation in the expression of TIMP-3 and Col-1a1, which increased the biomechanical properties of the ultimate stress and maximum load of healing tendon on a tendinopathy model. This provides evidence that the exosomes derived from tendon stem cells help balance the extracellular matrix of the tendon [54].

## Therapeutic potential of exosomes in tendinopathy

MSCs-exos are involved in a variety of biological phases of tendinopathy and have become a hot topic in the field of tendon healing (Table 1). In addition, several researches have focused on the exosomes-bearing scaffold, including collagen, gelatin, and hydrogel, for enhanced healing. A recent study examined how fibrin gel containing BMSCs-exos could boost exosome retention and stability in tendons, as well as mediate a dynamic remodeling process to speed tendon repair [49]. Hydrogel system is also a suitable carrier to deliver exosomes. BMSCs-exos-loaded hydrogel, for example, promote tendon-bone junction injury healing [55]. Although there is no current evidence on how this approach works in a model with clinical potential, success in larger mammal models may support clinical trials. Moreover, this raises the question of whether exosomes should directly contact the site of injury or be used in combination with other approaches to facilitate healing.

**Table 1:** MSCs-exos applications in tendinopathy

Exosomes	Animal model OR in vitro experiments	Delivery method	Mechanisms	Biological effects
BMSCs- exos	Rat patellar tendon defect model [49] Mouse tendon-bone reconstruction model [55] Rat medial collateral ligament (MCL) injury model [56]	Fibrin gel containing BMSCs-exos [49] Exosomes mixed with hydrogel [55] Exosome‐educated macrophages (EEMs) [56]	Promote the accumulation of CD146 + TSCs [49] Reduce M1 macrophages and proinflammatory factors (IL-1β and IL-6) in local tissues, increase M2 macrophages [55, 56]	Facilitate the proliferation and migration of TSCs [49] Decrease cell apoptosis, increase cell proliferation, reduce ECM deposition, and suppress excessive scar formation [55] Upregulate expression of collagen type I and III [56]
ADSCs- exos	Mouse Achilles tendon injury and repair model [10] Rabbit rotator cuff tears (RCTs) model [57] Rat massive rotator cuff tear (MRCT) model [58]	ADSCs EVs-loaded collagen sheet [10] ADSCs-exos in saline [57, 58]	Reduce NF‐κB activity, *Il1b* and *Ifng* expression; increase expression of Col1A1 and Col3A1 and thus decrease *Mmp1,* increase *Scx* and *Tnmd* [10]	Decrease muscle fatty infiltration [57] Promote anti-inflammatory, anti-apoptotic, and regenerative effects on rotator cuff tears [58] Modulate macrophage inflammatory response to reduce the early tendon inflammatory response after injury [10]
TSC-exos	Rat Achilles tendon injury model [47] Rat Achilles tendon tendinopathy model [54] Rat patellar tendon defect model [59]	TSC-exos mixed in gelatin methacryloyl (GelMA, EFL-GM-60, 10%w/v) [47] Exosome injection [54] Isolated sEVs-mixed sodium alginate hydrogel [59]	Activate PI3K/AKT and MAPK/ERK1/2 signaling pathways Increase the number of CD163 + and IL-10 + cells; decrease the number of CCR7 + , IL-6 + , and Cox-2 + cells [47] Decrease MMP-3 and α-SMA expression, increase TIMP-3 and Col-1a1 [47, 54] Activate yes-associated protein (YAP) via the H19-PP1-YAP axis [59]	Promote effective healing of injured tendons [47] Increase capacity of biomechanical properties of ultimate stress and maximum loading [54] Promote proliferation, differentiation, migration, collagen deposition, and YAP localization [59]
Purified exosome product (PEP) derived from plasma	In vitro experiments on dog flexor tendon [60, 61]	PEP exosomes [60, 61]	Increase expression of genes linked to tendons (SCX, COL1A, COL3A1, TNMD, DCN, and MKX) [61] Increase expression of COL3A1, MMP2, MMP3, and MMP14; reduce expression of IL‐6 and TGF‐β [60]	Maintain capability of tenocyte migration and increase total collagen deposition, attenuate dexamethasone‐induced cellular apoptosis [61] Reduce inflammation and improve type III collagen expression [60]
HUMSC-derived exosomes	Rat Achilles tendon injury adhesion model [62]	HUMSC-exos dissolved in PBS [62]	Deliver low-abundance miR-21a-3p, thus control p65 activity [62]	Reduce fibroblast proliferation and inhibit the expression of fibrosis genes: collagen III (COL III) and α-smooth muscle actin (α-SMA) in vitro [62] Relieve tendon adhesion [62]

Until the underlying mechanisms of biological behaviors of exosomes are better understood, it will not be possible to design exosomes with specific functions, such as exosomes that can act as messengers to regulate the characteristics of different cells or function as a diagnostic biomarker to reveal the progression of various diseases [63]. Several studies have examine how exosomes can serve as both natural and engineered nanocarriers for delivering drug molecules, nucleic acids, and proteins for therapeutic purposes.

Exosomes could serve as a delivery systems to deliver inflammatory factor antagonists or miRNA for the treatment of tendon diseases. Their inherent biochemical properties give exosomes great potential as a reliable drug delivery system for targeted therapy [64, 65].

Adhesive proteins within the lipid bilayer and cytosol enable exosomes to pair with ligands on recipient cell surfaces for targeted therapy and effective protein distribution [66]. Exosome-based therapeutics based on advanced nanotechnology has shown promise for the treatment of tendinopathy. In a recent study, Yao et al. applied antagonists targeting human miR-21a-3p (fibrosis stimulate factor) to human umbilical cord mesenchymal stem cells (HUMSCs) and obtained functional exosomes with enhanced inhibition of tendon adhesion [62]. Given this result, it is convinced that the sustained exploration of advanced design methodologies, traditional nanomedicine, or novel gene therapy with exosomes will improve treatment prospects for tendon healing.

## Conclusion

Exosomes never fail to fascinate researchers. They are indispensable to physiological and pathophysiological processes, yet much about them is unknown and remains to be investigated. This review has taken insight from the biogenesis and function of MSC-exos to present the chance for use of exosomes in the treatment of tendinopathy. Specifically, the potential functions for MSCs-exos in the diagnosis and treatment of tendinopathy were reported. Although insufficient investigation has been done into the possible mechanisms of MSC-exos for tissue regeneration, the information that has been revealed suggests the possibility of MSC-exos being helpful in the treatment and diagnosis of tendinopathy.

## Data Availability

Not applicable.

## Abbreviations

MSCs: Mesenchymal stem cells
MSCs-exos: MSCs exosomes
MMPs: Matrix metalloproteinases
ADSCs: Adipose-derived stem cells
ILVs: Intraluminal vesicles
ESCRT: Endosomal sorting complex required for transport
MVBs: Multivesicular bodies
GTPase: Guanosine triphosphatase
TSCs: Tendon stem cells
EVs: Extracellular vehicles
RC: Rotator cuff
TSCs-exos: Tendon stem cells-derived exosomes
EEMs: Exosome‐educated macrophages
BMSCs-exos: Bone marrow mesenchymal stem cells-derived exosomes
TGF: Transforming growth factor
ECM: Extracellular matrix
TIMP: Tissue inhibitors of metalloproteinases
MCL: Rat medial collateral ligament
RCTs: Rabbit rotator cuff tears
MRCT: Rat massive rotator cuff tear
YAP: Yes-associated protein
α-SMA: α-Smooth muscle actin
HUMSCs: Human umbilical cord mesenchymal stem cells
